# Supplementary material for: Genetic prediction with ARG-powered linear algebra
Source: Genetics. 2026 Mar 27;233(1):iyag074. doi: 10.1093/genetics/iyag074 (PMC13147536; doi:10.1093/genetics/iyag074)
Supplement: iyag074_Supplementary_Data [file iyag074_supplementary_data.pdf]

Appendix

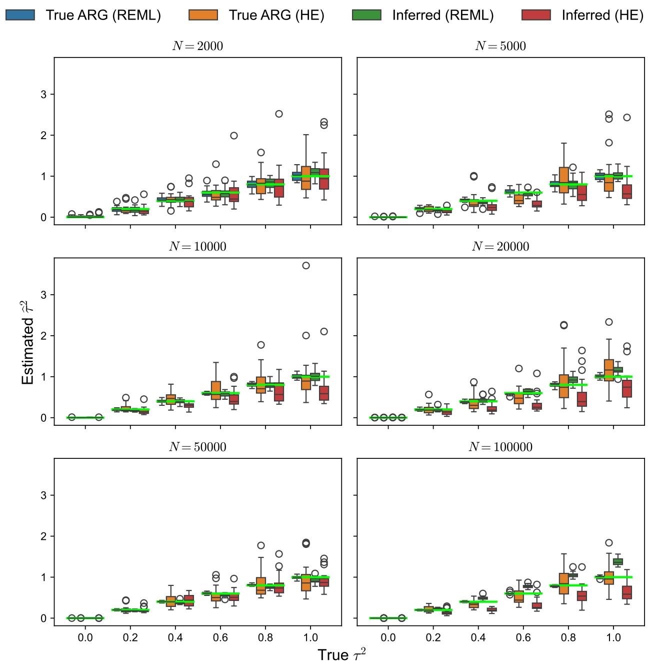

Figure A1 Statistical precision of the HE method and AI-REML. By default, the branch GRM is scaled by 1 over $4\sum_T s_T t_{\text{root},T}$. The values in the labels "HE (value)" refer to the additional scaling we apply to the GRM. Each boxplot shows the distribution of estimators over 100 replicates.

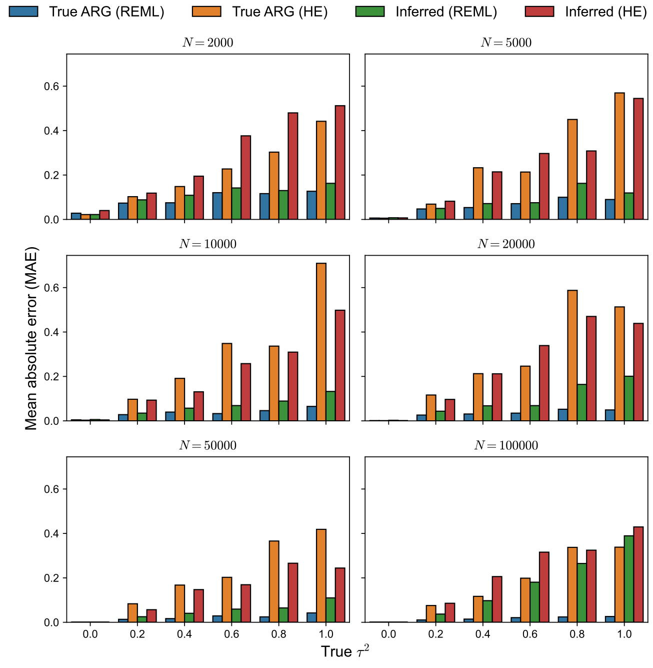

Figure A2 Statistical precision of the HE method and AI-REML. By default, the branch GRM is scaled by 1 over $4\sum_T s_T t_{\text{root},T}$. The values in the labels "HE (value)" refer to the additional scaling we apply to the GRM. The mean absolute error (MAE) of the estimators with respect to the true parameters $\sigma_\varepsilon^2 = 1$ and $\tau^2 = 1$.

Model formulation and assumptions

Expressing a complex trait on an ARG

We show the correspondence between site and branch (edge) based trait expressions. We define an ARG $\mathcal{T} = (\mathcal{N}, \mathcal{E})$ as a collection of nodes $\mathcal{N}$ and edges $\mathcal{E}$. Each node n has a (birth) time ago t_n, and each edge e indicates inheritance of a genomic interval $[\ell_e, r_e)$ by a child $c(e)$ from a parent $p(e)$ (Wong *et al.* 2024). We call these "parent" and "child" in graph terms, so the inheritance might be over more than one generation. To describe a valid ARG, the spans of all edges with the same child must be disjoint, and the time of the parent must be larger than the time of the child for each edge. We will also call the *span* of an edge $s_e = r_e - \ell_e$ (in number of base-pairs), and the *length* of an edge $l_e = t_{p(e)} - t_{c(e)}$ (in time units, such as generations). All expectations and variances are conditioned on $\mathcal{T}$, which accounts for the variation in observed genome data due to recombination and drift, and hence also the implicit population structure and pedigree of individuals in $\mathcal{T}$. Only the mutational process is left random in this setting. This setting was explored in Ralph (2019) and expanded in Ralph *et al.* (2020). In those papers, the utility of treating mutations as random on a fixed ARG was to understand how well a given SNP genotype (site) statistic could be expected to estimate the corresponding summary of tree shapes.

For conceptual clarity, we assume in the following that the edges are in fact *sub-edges*: each has a unique sub-topology along its span. (Although this is not true in the case of tree sequences in general, we can enforce this by appropriate splitting of edges as in Salehi Nowbandegani *et al.* (2023).) Nevertheless, this splitting is merely conceptual and never takes place in the algorithms we'll demonstrate shortly, as it significantly degrades the storage efficiency of the tree sequence data structure.

We consider complex traits that follow a linear model. Write $\mathbf{G} \in \mathbb{R}^{N \times P}$ for the $N \times P$ genotype matrix for N genomes and P sites in the genome, whose $(i, j)^{\text{th}}$ entry is the number of derived alleles carried by the ith genome at the jth site. Then the trait model is:

$$\mathbf{y} = \mathbf{G}\boldsymbol{\beta} + \boldsymbol{\varepsilon} = \sum_{p=1}^{P} \mathbf{G}_p \boldsymbol{\beta}_p + \boldsymbol{\varepsilon}, \tag{A1}$$

where $\boldsymbol{\beta} \in \mathbb{R}^P$ is a vector defining a effect size for the derived allele(s) at each site. For now, we condition on $\boldsymbol{\beta}$ so that we treat it as a fixed quantity. With infinite-sites mutation model, each site has at most two alleles, so this effect is the allele substitution effect (Falconer 1995), also called the site effect, variant effect, or mutation effect in this work. One cannot estimate $\boldsymbol{\beta}$'s entries separately because some variants appear on the same edge (or on the two edges pendant to the root). Columns of $\mathbf{G}$ corresponding to these variants will be identical, leading to perfect collinearity (linkage-disequilibrium), making them statistically indistinguishable. We will group these indiscernible variants according to their edge membership. After conditioning on $\mathcal{T}$, the remaining variation in $\mathbf{G}$ is solely due to the mutational process. We assume that mutations occur on edge e following a Poisson process at a rate proportional to the total number of base-pairs (span) multiplied by the amount of time the edge represents (length), $l_e s_e$. We let the per unit time per base-pair mutation rate u_{ep} vary across edges and positions If we let $\mathbf{1}_{ep}$

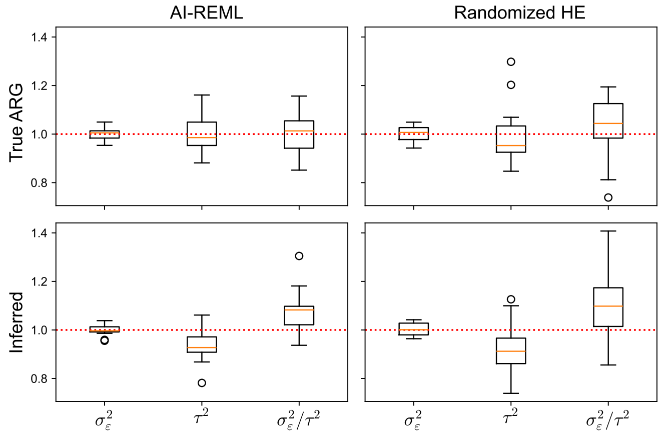

Figure A3 Statistical precision of the HE method and AI-REML in the spatial simulation benchmark. 20 replicate experiments were conducted.

be an indicator variable that is 1 if a mutation has arrived on edge e at position p and 0 otherwise, $\mathbb{E}\left[\mathbf{1}_{ep}\right] = l_e u_{ep}$.

Recurrent mutations are allowed; however, mutations cannot occur below an existing one. This is a slight relaxation of the infinite-sites model to allow multiple recurrent mutations in unrelated lineages but no back mutation. Define $\mathbf{Z} \in \mathbb{R}^{N \times E}$ so that $\mathbf{Z}_{ie}$ is the number of chromosomes of individual i that is a descendant of edge e. $\mathbf{Z}_{ie}$ ranges from 0 to 2 in the case of diploids. We can express $\mathbf{G}$ with $\mathbf{Z}$ by:

$$\mathbf{G}_{ip} = \sum_{e:p \in e} \mathbf{Z}_{ie} \mathbf{1}_{ep}, \tag{A2}$$

where $p \in e$ means edge e contains the position p, and define $\mathbf{G}_p$ as the p-th column of $\mathbf{G}$. In words, one can determine individual i's genotype at position p by first examining if edge e is ancestral to i ($\mathbf{Z}_{ie}$) and then checking if the edge has a mutation at p ($\mathbf{1}_{ep}$). Finally, we sum up such contributions across all edges ($\sum_{e:p\in e}$). This also illustrates how `tstrait` computes the trait values: for a position p in a local tree, it travels down the edges (or equivalently branches for now) of the tree and checks if there's a mutation along the path from the root to the tips. Here, $\mathbf{Z}_{ie}$ tells if an edge in $\{e : p \in e\}$ is on the path from the root to a tip, which is individual i.

Using equation (A2), we can collapse variants corresponding to perfectly correlated columns of $\mathbf{G}$ into the edge on which they reside. This is a natural choice because mutations on the same edge are inherited by the same set of individuals (recall that the edge's subtree is constant by assumption). Substituting (A2) in the linear equation (A1) gives:

$$\begin{aligned}
\mathbf{y} &= \sum_{p=1}^{P} \mathbf{G}_p \boldsymbol{\beta}_p + \boldsymbol{\varepsilon} \\
&= \sum_{p=1}^{P} \left(\sum_{e:p\in e} \mathbf{Z}_e \boldsymbol{\beta}_p \mathbf{1}_{ep} \right) + \boldsymbol{\varepsilon} \\
&\because \text{substitute (A2)} \\
&= \sum_{e=1}^{E} \sum_{p:p\in e} \mathbf{Z}_e \boldsymbol{\beta}_p \mathbf{1}_{ep} + \boldsymbol{\varepsilon} \\
&\because \text{swap the sums over } e \text{ and } p \\
&= \sum_{e=1}^{E} \mathbf{Z}_e \left(\sum_{p:p\in e} \boldsymbol{\beta}_p \mathbf{1}_{ep} \right) + \boldsymbol{\varepsilon} \\
&\because \mathbf{Z}_e \text{ does not depend on } p \\
&= \sum_{e=1}^{E} \mathbf{Z}_e \mathbf{v}_e + \boldsymbol{\varepsilon} \quad \because \mathbf{v}_e = \sum_{p:p\in e} \boldsymbol{\beta}_p \mathbf{1}_{ep} \\
&= \mathbf{Z}\mathbf{v} + \boldsymbol{\varepsilon}.
\end{aligned} \tag{A3}$$

The key step is at the third line which swaps the summation over edges e and sites p. Then, we pulled out $\mathbf{Z}_e$ out of the inner sum over p and merged the inner sum into $\mathbf{v}_e$. This derivation presents a way to convert a genotype-based expression of a trait $\mathbf{y} = \sum_p \mathbf{G}_p \boldsymbol{\beta}_p + \boldsymbol{\varepsilon}$ into a branch-based expression $\mathbf{y} = \sum_e \mathbf{Z}_e \mathbf{v}_e + \boldsymbol{\varepsilon}$.

Linear algebra formulation

We now connect this linear model to classical formulations in quantitative genetics and selective breeding literature on the pedigree model (Henderson 1976; Quaas 1988). While we work with a linear model on an ARG, not pedigree, our aim here is to highlight the inherent sparse structure of phenotype generative models and the critical connection with sparse linear algebra and efficient computational algorithms (Rue and Held 2005). We start by noting that sub-edges are also edges – each has a parent and child node and a genomic interval – but with the property (as above) that the subtree below them in the ARG does not change. These can be obtained as follows: iterating along the genome, each time the local tree changes, it changes through addition or removal of an edge. If the parent of the newly added or removed edge is n, then we move up through the local tree, and split each edge we encounter. For instance, if n is not a root then there is an edge with child n and parent p, and interval $[\ell, r)$ that spans the current position x. By "splitting" this edge we mean to replace it with two edges: one with interval $[\ell, x)$ and the other with interval $[x, r)$. The resulting shorter edges will be all sub-edges.

We say that sub-edge e_1 *directly inherits* from sub-edge e_2 if $p(e_1) = c(e_2)$ and their genomic intervals overlap. In fact, it then follows that the interval of e_2 is contained within that of e_1: in other words, $[\ell(e_2), r(e_2)) \subseteq [\ell(e_1), r(e_1))$. Now, define the square matrix $\mathbf{A}$ to have rows and columns indexed by sub-edges, with $\mathbf{A}_{ef} = 1$ if sub-edge e directly inherits from f, and 0 otherwise. This $\mathbf{A}$ is $\mathbf{P}$ in Quaas (1988). The *sub-edge inheritance matrix* is then $\mathbf{I} + \mathbf{A} + \mathbf{A}^2 + \cdots = (\mathbf{I} - \mathbf{A})^{-1}$: this has $(e, f)^{\text{th}}$ entry equal to 1 if $e = f$ or if e inherits from f (i.e., if the edge represented by e carries genetic material inherited also along f), and 0 otherwise. Note that the seemingly infinite series above is in fact a finite sum because the depth of an ARG is finite. Now, write $\mathbf{M}$ for the *individual-sub-edge incidence matrix*: for an

individual i and a sub-edge e, let $\mathbf{M}_{ie} = 1$ if $c(e) \in i$ and $\mathbf{M}_{ie} = 0$ otherwise. $c(e) \in i$ means individual i contains node $c(e)$. The matrix $\mathbf{Z}$ from the main text is thus:

$$\mathbf{Z} = \mathbf{M}(\mathbf{I} - \mathbf{A})^{-1}.$$

The directed graph whose adjacency matrix is $\mathbf{A}$, the *sub-edge graph*, is a multitree, and has many convenient properties. For instance, many standard ARG computations that require left-to-right iteration can be implemented as bottom-up or top-down on the sub-edge graph. This property is shared by the genome representation graph of DeHaas *et al.* (2024). Typically, there will be a multiplicative factor of order $\log N$ more sub-edges than edges, where N is the sample size. However, this difference can be large in practice as the constant depends on demographic history. An advantage to working with the sub-edge graph is that it allows use of more general graph-based tools, as for instance by Salehi Nowbandegani *et al.* (2023). However, we are not aware of a reason that such algorithms are necessarily more efficient.

This formulation is useful conceptually, but we did not find it to be useful computationally, because typical left-to-right algorithms on the ARG naturally work with sub-edges without explicitly constructing them (for instance, those in Ralph *et al.* (2020) or Zhu *et al.* (2025)). We are not aware of any argument that algorithms using a structure like the sub-edge graph are inherently more efficient than algorithms that efficiently use the tree sequence.

Sub-edges, and the "splitting" procedure are very similar to the "bricks" and the "bricking" operation used by Salehi Nowbandegani *et al.* (2023). However, "bricks" are defined so that they have a unique set of descendant nodes, rather than a unique subtree. Since subtrees can change but still have the same set of descendants, bricks are thus larger than sub-edges, while retaining the useful properties above. However, the brick graph is no longer a multitree. We chose the more strict definition mainly because it does not require retaining and checking the descendant nodes at each step.

The linear algebra formulation shows the sparse structure of our trait generative model on an ARG, enabling scalable algorithms as shown in Lehmann *et al.* (2025) and this study. For completeness, we point to Misztal (2015), who followed a similar goal – enabling inexpensive computations with the inverse of genotype GRM by avoiding inversion of a large GRM. Instead of focusing on the trait generative model, Misztal (2015) split the genotyped sample into core and non-core sets of individuals and expressed genetic values of non-core individuals conditional on core individuals. This conditioning also admits a sparse inverse of genotype GRM and is technically the same as conditioning on "inducing point" or "knots" in spatial modeling (e.g., Heaton *et al.* 2018). While this core conditioning approach is computationally very efficient for populations with limited genetic diversity, such as in selective breeding, it does not scale as well to more diverse settings as the work presented in this study.

The branch-based phenotype model is a linear mixed model

The last expression we arrived at expresses a phenotype in terms of edges:

$$\mathbf{y} = \sum_{e} \mathbf{Z}_e \mathbf{v}_e + \boldsymbol{\varepsilon}. \tag{A4}$$

We refer to $\mathbf{v}_e$ as the edge effect and we shall characterize its probabilistic features such as its distribution. We go back to the definition of the edge effect and observe that it is a collection of indicator random variables driven by mutation:

$$\mathbf{v}_e = \sum_{p:p\in e} \boldsymbol{\beta}_p \mathbf{1}_{ep}. \tag{A5}$$

In turn, $\mathbf{v}_e$ as a whole is a random variable. Since mutations occur at a low rate, $\mathbf{v}_e$ is likely to be zero most of the time, but conditioning on the occurrence, a mutation hits position p at a probability that is proportional to the position's mutation rate u_{ep}. The effect size of this mutation is $\boldsymbol{\beta}_p$. Altogether, $\mathbf{v}_e$ is zero or a number drawn from the pool of effect sizes $\{\boldsymbol{\beta}_p\}_{p\in e}$ with probability proportional to the mutation rate u_{ep}. Hence, the distribution is determined by the collection $\{(\boldsymbol{\beta}_p, u_{ep})\}_{p:p\in e}$.

This formulation closely mirrors the continuum-of-alleles (CoA) model proposed by Kimura and Crow (1964). Similar to an edge that contains many positions, a locus also stretches over a genome segment that is subject to mutation. When a mutation arrives, it occurs at a random position yielding a different allele every time, i.e., the infinite-alleles model. The CoA model describes the distribution of the effect size of these newly emerging alleles.

Our branch based expression in equation (A4) represents a phenotype by a design matrix with effect sizes that are random with respect to mutations. Hence, it can be considered as a linear random effects model. Furthermore, some features of the model can be deduced from the properties of the mutational process and the topology of the ARG. The variance of an edge can be computed as follows:

$$\begin{aligned}
\mathrm{Var}\left(\mathbf{v}_e\right) &= \mathrm{Var}\left(\sum_{p:p\in e} \boldsymbol{\beta}_p \mathbf{1}_{ep}\right) \\
&= \sum_{p:p\in e} \boldsymbol{\beta}_p^2 \mathrm{Var}\left(\mathbf{1}_{ep}\right) \\
&\quad \because \text{mutations at different } p \text{ are independent} \\
&= \sum_{p:p\in e} \boldsymbol{\beta}_p^2 l_e u_{ep}(1 - l_e u_{ep}) \\
&\quad \because \mathrm{Var}\left(\mathbf{1}_{ep}\right) = \mathbb{E}\left[\mathbf{1}_{ep}\right]\left(1 - \mathbb{E}\left[\mathbf{1}_{ep}\right]\right) \\
&\approx \sum_{p:p\in e} \boldsymbol{\beta}_p^2 l_e u_{ep} \qquad\qquad \text{(A6)} \\
&\quad \because l_e u_{ep} \ll 1 \text{ i.e., mutation rate is small} \\
&= \overbrace{l_e s_e}^{\text{edge area}:=A_e} \cdot \overbrace{\frac{1}{s_e} \sum_{p:p\in e} \boldsymbol{\beta}_p^2 u_{ep}}^{\substack{\text{size average of squared} \\ \text{effect sizes times mutation} \\ \text{rate}:=\tau_e^2}} \\
&\quad \because l_e \text{ does not depend on } p \\
&= A_e \tau_e^2
\end{aligned}$$

The variance of an edge effect is proportional to its area A_e. τ_e^2 is the average squared effect size weighted by mutation rates. In words, regions with higher functional importance (large $\boldsymbol{\beta}_p^2$) and mutation rate (large u_{ep}) contribute more to branch/edge effect variation. Assuming that mutation rates are constant and the effect sizes are evenly dispersed across the genome, similar to the infinitesimal model, this recovers the ARG-LMM model we have used in the main portion of this paper.

In the main portion of the paper, the branch effects were assumed to be mean zero and mutually independent. A derivation

of these (approximate) properties helps shed light on how and when this is a good assumption. To do this, we decompose $\mathbf{v}_e$ into two parts $\mathbf{u}_e = \mathbf{v}_e - \mathbb{E}[\mathbf{v}_e]$ and $\mathbf{f}_e = \mathbb{E}[\mathbf{v}_e]$. The vector of these quantities are defined accordingly: $\mathbf{u} = \mathbf{v} - \mathbb{E}[\mathbf{v}]$ and $\mathbf{f} = \mathbb{E}[\mathbf{v}]$. Then, we can rewrite equation (A4) as:

$$\mathbf{y} = \mathbf{Zf} + \mathbf{Zu} + \varepsilon. \tag{A7}$$

Henceforth, we refer to $\mathbf{u}_e$ instead of $\mathbf{v}_e$ as the edge effect. From the definition, the branch effect $\mathbf{u}_e$ has mean zero. At first glance, enforcing zero mean by subtracting the mean looks like an unfounded assumption that sweeps the mean contributions $\mathbf{Zf}$ under the rug. Surprisingly, if all individuals exist at the same time, this is not an issue. Under neutral evolutionary scenarios of various kinds, we may assume that the mutation rate is constant for each position (Ewens 2004; Wakeley 2008; Durrett 2008), i.e., $u_{ep} = u_p$ for all e such that $p \in e$ (edge e contains position p). In this case, $\mathbf{Zf}$ reduces to a constant:

$$\begin{aligned}
[\mathbf{Zf}]_i &= \sum_{e=1}^{E} \mathbf{Z}_{ie} \mathbb{E}\left[\sum_{p:p\in e} \boldsymbol{\beta}_p \mathbf{1}_{ep}\right] \\
&\because \mathbf{f}_e = \mathbb{E}[\mathbf{v}_e] \\
&= \sum_{e=1}^{E} \sum_{p:p\in e} \mathbf{Z}_{ie} \boldsymbol{\beta}_p l_e u_p \\
&\because \mathbb{E}\left[\mathbf{1}_{ep}\right] = l_e u_{ep} \text{ and } u_{ep} = u_p \\
&= \sum_{p=1}^{P} \sum_{e:p\in e} \mathbf{Z}_{ie} \boldsymbol{\beta}_p l_e u_p \\
&\because \text{swap the sums over } e \text{ and } p \\
&= \sum_{p=1}^{P} \boldsymbol{\beta}_p u_p \left(\sum_{e:p\in e} \mathbf{Z}_{ie} l_e\right) \\
&\because \boldsymbol{\beta}_p \text{ and } u_p \text{ do not depend on } e \\
&= \sum_{p=1}^{P} \boldsymbol{\beta}_p u_p \cdot 2 t_{\text{root},p} \quad \because \text{ploidy} = 2 \\
&= \text{constant with respect to } i.
\end{aligned} \tag{A8}$$

Note that $\sum_{e:p\in e} \mathbf{Z}_{ie} l_e$ is the sum of lengths of all edges ancestral to individual n that adds up to the root time at position p multiplied by ploidy. This means that the fixed component of the genetic effect is fully accounted for by a model intercept.

We can further generalize equation (A8) to incorporate time varying mutation rates. In this setting, we stipulate that a mutation rate at a position only depends on time, i.e., the mutation rate is a function of time $u_p(t)$:

$$\begin{aligned}
[\mathbf{Zf}]_i &= \sum_{e=1}^{E} \mathbf{Z}_{ie} \mathbb{E}\left[\sum_{p:p\in e} \boldsymbol{\beta}_p \mathbf{1}_{ep}\right] \\
&= \sum_{e=1}^{E} \sum_{p:p\in e} \mathbf{Z}_{ie} \boldsymbol{\beta}_p \mathbb{E}\left[\mathbf{1}_{ep}\right] \\
&\because \mathbf{Z}_{ie} \text{ does not depend on } p \\
&= \sum_{e=1}^{E} \sum_{p:p\in e} \mathbf{Z}_{ie} \boldsymbol{\beta}_p \int_{t(e(c))}^{t(e(p))} u_p(t)dt \\
&\because \mathbb{E}\left[\mathbf{1}_{ep}\right] = \int_{t(e(c))}^{t(e(p))} u_p(t)dt \\
&= \sum_{p=1}^{P} \sum_{e:p\in e} \mathbf{Z}_{ie} \boldsymbol{\beta}_p \int_{t(e(c))}^{t(e(p))} u_p(t)dt \\
&\because \text{swap the sums over } e \text{ and } p \\
&= \sum_{p=1}^{P} \boldsymbol{\beta}_p \sum_{e:p\in e} \mathbf{Z}_{ie} \int_{t(e(c))}^{t(e(p))} u_p(t)dt \\
&\because \boldsymbol{\beta}_p \text{ does not depend on } e \\
&= \sum_{p=1}^{P} \boldsymbol{\beta}_p \left(2 \int_{0}^{t_{\text{root},p}} u_p(t)dt\right) \quad \because \text{ploidy} = 2 \\
&= \text{constant. w.r.t. } i.
\end{aligned} \tag{A9}$$

This confirms that the fixed portion $\mathbf{Zf}$ of equation (A7) is constant in a fairly general setting with time varying mutation rates.

In order to demonstrate independence, we show that $\text{Cov}(\mathbf{u}_e, \mathbf{u}_{e'}) = 0$ for all pairs $e \neq e'$. This implies independence because indicator variables are independent if and only if they are uncorrelated, similar to Gaussian random variables. We first expand $\text{Cov}(\mathbf{u}_e, \mathbf{u}_{e'})$ and reduce it to a sum of covariances of indicators:

$$\begin{aligned}
&\text{Cov}(\mathbf{u}_e, \mathbf{u}_{e'}) \\
&= \text{Cov}\left(\sum_{p:p\in e} \boldsymbol{\beta}_p \mathbf{1}_{ep}, \sum_{p':p'\in e'} \boldsymbol{\beta}_{p'} \mathbf{1}_{e'p'}\right) \\
&= \sum_{p\in e, p'\in e'} \boldsymbol{\beta}_p \boldsymbol{\beta}_{p'} \text{Cov}\left(\mathbf{1}_{ep}, \mathbf{1}_{e'p'}\right) \\
&\because \text{Cov is bilinear} \\
&= \sum_{p\in e,e'} \boldsymbol{\beta}_p^2 \text{Cov}\left(\mathbf{1}_{ep}, \mathbf{1}_{e'p}\right) \\
&\because \text{mutations at different } p \text{ are independent.}
\end{aligned} \tag{A10}$$

The last line's simplification follows from the independence of indicator variables $\mathbf{1}_{ep}$ across different positions. When e and e' have no overlapping positions, the summation is trivially zero as $\{p : p \in e, e'\}$ will be an empty set. It's also zero if the two edges are not ancestral to each other, as mutations running on

separate edges are also independent. Otherwise:

$$\begin{aligned}
&\mathrm{Cov}\left(\mathbf{1}_{ep}, \mathbf{1}_{e'p}\right)\\
&= \mathbb{E}\left[\mathbf{1}_{ep}\mathbf{1}_{e'p}\right] - \mathbb{E}\left[\mathbf{1}_{ep}\right]\mathbb{E}\left[\mathbf{1}_{e'p}\right]\\
&\because \mathrm{Cov}\left(X,Y\right) = \mathbb{E}\left[XY\right] - \mathbb{E}\left[X\right]\mathbb{E}\left[Y\right]\\
&= \mathbb{E}\left[\mathbb{E}\left[\mathbf{1}_{ep}\mathbf{1}_{e'p} \mid \mathbf{1}_{ep}\right]\right] - l_e u_{ep} l_{e'} u_{e'p}\\
&\because \mathbb{E}\left[X\right] = \mathbb{E}\left[\mathbb{E}\left[X \mid Y\right]\right]\\
&= \mathbb{E}\left[\mathbf{1}_{ep}\mathbb{E}\left[\mathbf{1}_{e'p} \mid \mathbf{1}_{ep}\right]\right] - l_e u_{ep} l_{e'} u_{e'p}\\
&\because \mathbb{E}\left[XY \mid X\right] = X\mathbb{E}\left[Y \mid X\right]\\
&= \mathbb{P}\left(\mathbf{1}_{ep} = 0\right)\cdot 0 \cdot l_{e'} u_{e'p}\\
&+ \mathbb{P}\left(\mathbf{1}_{ep} = 1\right)\cdot 1 \cdot 0 - l_e u_{ep} l_{e'} u_{e'p}\\
&= -l_e u_{ep} l_{e'} u_{e'p}.
\end{aligned} \tag{A11}$$

This latter quantity is small because it is proportional to the per base-pair mutation rate squared. Not allowing child mutations gives $\mathbb{E}\left[\mathbf{1}_{e'p} \mid \mathbf{1}_{ep} = 1\right] = 0$, provided e is an ancestor of e' without the loss of generality. Therefore, we can justify the diagonal covariance matrices of random effects $\mathbf{u}$ by setting all terms involving u_{ep}^2 to zero.

In summary, we have the resulting generalization, that allows varying mutational variance along the genome and through time: The distribution of a complex trait follows a linear mixed model conditioned on the sample's ARG $\mathcal{T} = (\mathcal{N}, \mathcal{E})$:

$$\mathbf{y} = \mathbf{u}_0 + \mathbf{Z}\mathbf{u} + \boldsymbol{\varepsilon}, \tag{A12}$$

with a constant vector $\mathbf{u}_0$ (all entries have the same value). The random effects $\mathbf{u}$'s covariance matrix is:

$$\boldsymbol{\Sigma}_{\mathbf{u}} = \mathrm{diag}\left[l_e s_e \tau_e^2\right]_{e=1,\ldots,E}, \tag{A13}$$

where $\tau_e^2 = \frac{1}{s_e}\sum_{p:p\in e}\beta_p^2 u_{ep}$. The residual $\boldsymbol{\varepsilon}$ contains the contribution of non-genetic variables. We call the covariance matrix $\mathrm{Var}\left(\mathbf{Z}\mathbf{u}\right) = \mathbf{Z}\boldsymbol{\Sigma}_{\mathbf{u}}\mathbf{Z}^T$ as the generalized branch GRM. In the model we use in the main paper, we further assume that $\boldsymbol{\beta}_p \sim \mathrm{Normal}(\boldsymbol{\mu}_p, \tau^2)$. Then, $\mathrm{Var}\left(\mathbf{Z}\mathbf{u}\right)$ further reduces to

$$\mathrm{Var}\left(\mathbf{Z}\mathbf{u}\right) = \tau^2\mathbf{B} \tag{A14}$$

in which $\mathbf{B}$ is the branch GRM from Lehmann *et al.* (2025). $\boldsymbol{\mu}_p$ does not affect the GRM and is absorbed to the intercept as in equation (A8) and (A9).

Variance of V_G

First, note that if X and Y are Gaussian, then Isserlis' Theorem (Isserlis 1918) says that:

$$\mathbb{E}\left[X^2Y^2\right] = \mathbb{E}\left[X^2\right]\mathbb{E}\left[Y^2\right] + 2\mathbb{E}\left[XY\right]^2.$$

Expanding out $\mathrm{Var}\left(V_G\right)$, we will use this fact with $X = (\mathbf{P}_N\mathbf{g})_n$ and $Y = (\mathbf{P}_N\mathbf{g})_m$. Continuing with the notation from equation (18):

$$\begin{aligned}
&\mathrm{Var}\left(V_G\right)\\
&= \mathrm{Var}\left(\frac{1}{N}\sum_{n=1}^{N}(\mathbf{g}_n - \overline{\mathbf{g}})^2\right)\\
&= \frac{1}{N^2}\left(\mathbb{E}\left[(\mathbf{P}_N\mathbf{g})^T(\mathbf{P}_N\mathbf{g})(\mathbf{P}_N\mathbf{g})^T(\mathbf{P}_N\mathbf{g})\right] - \mathbb{E}\left[(\mathbf{P}_N\mathbf{g})^T(\mathbf{P}_N\mathbf{g})\right]^2\right)\\
&= \frac{1}{N^2}\sum_{n=1}^{N}\sum_{m=1}^{N}\left(\mathbb{E}\left[(\mathbf{P}_N\mathbf{g})_n^2(\mathbf{P}_N\mathbf{g})_m^2\right] - \mathbb{E}\left[(\mathbf{P}_N\mathbf{g})_n^2\right]\mathbb{E}\left[(\mathbf{P}_N\mathbf{g})_m^2\right]\right)\\
&= \frac{1}{N^2}\sum_{n=1}^{N}\sum_{m=1}^{N}\mathbb{E}\left[(\mathbf{P}_N\mathbf{g})_n(\mathbf{P}_N\mathbf{g})_m\right]^2\\
&= \tau^4\frac{1}{N^2}\sum_{n=1}^{N}\sum_{m=1}^{N}\left(\widetilde{\mathbf{B}}_{n,m}\right)^2.
\end{aligned} \tag{A15}$$

Since the offdiagonals of $\widetilde{\mathbf{B}}$ are $\mathcal{O}(1/N)$ (where recall that N is the sample size), the last line tells us that $\mathrm{Var}\left(V_G\right) = \mathcal{O}(1/N)$, and hence, $\tau^2\,\mathrm{tr}(\widetilde{\mathbf{B}})/N$ is a consistent estimator of V_G, with an error that is of order $1/\sqrt{N}$.

Correctness of Algorithm T

Here we present an algorithm for efficient simulation from the ARG-LMM model. This serves several purposes: first, the structure of the algorithm helps to highlight the dependency structures within the ARG. Second, the simplest tree-by-tree trait simulation algorithm for a large ARG could be computationally very costly, and this algorithm is much more efficient. Third, to illustrate how a key part of the matrix-vector product algorithm in Lehmann *et al.* (2025) works.

We begin with a precise explanation of what we'd like to simulate. Consider a given tree T that extends over some span s_T of the genome, and for an edge or sub-edge e and a node n write $e \geq_T n$ if e is ancestral to n in T. Each edge $e \in T$ connects a child $c(e)$ to a parent $p(e)$, and so has area $s_T(t_{p(e)} - t_{c(e)})$ in this tree. For each edge in each tree, let the edge effect $\mathbf{u}_{e,T}$ be a Gaussian random variable with mean zero and variance $s_T(t_{p(e)} - t_{c(e)})$. Then, the genetic value of a node n – the quantity we seek to simulate – is equal to $\sum_T\sum_{e\geq_T n}\mathbf{u}_{e,T}$. However, if the subtree below an edge e does not change across trees $T_i, T_{i+1}, \ldots, T_j$, then these effects can be combined: $\mathbf{u}_{e,T_i} + \cdots + \mathbf{u}_{e,T_j}$ is inherited together by the same set of nodes. By additivity of Gaussian distributions, this combined effect is itself Gaussian with variance equal to the combined areas, and so we aim to simulate $\mathbf{Z}\mathbf{u}$, where $\mathbf{Z}$ is the individual–sub-edge design matrix defined above and $\mathbf{u}$ is a vector of sub-edge effects from a Gaussian distribution with mean zero variance equal to sub-edge areas. (We emphasize that that in practice a single edge in the ARG can subtend many different subtrees, so that each e denotes a sub-edge as defined above.)

The algorithm works, roughly, as follows. The "value" $v(n)$ of each node caches a total contribution to genetic value that is inherited by every node in the current subtree below n. The "current value" of node n is obtained by summing up the values of all nodes above n in the current tree – so, if the tree changes, we must make adjustments to maintain a correct current value. When an edge with parent p and child c is removed from the tree, two things happen. First, the effect of that edge is simulated, and added to $v(c)$. Then, the value of p and every node above p must be added to $v(c)$, because c is no longer below

those nodes. Adding an edge creates a different problem: after adding the edge, c is below p in the tree, but $v(p)$ contains effects from previous parts of the genome. Therefore, we subtract the values of p and all nodes above p from $v(c)$, so that these effects cancel out when summing up the tree from c. At the end of the algorithm all edges are removed, so we end up with the empty tree, and so $v(n)$ will be equal to the genetic value of node n.

Algorithm T. (*Trait genetic value simulation*). Given a sequence of positions $0 = b_0 < b_1 < \cdots < b_K = L$ that are recombination breakpoints along the genome of length L, and corresponding sequences of edges to remove (R_k) and add (A_k) at each position, sample the genetic values g_s for $1 \leq s \leq n_S$, as long as all samples are leaves in all trees. Let T be the current tree, $\ell_T(n) = t_{p(n)} - t_n$ be the length of the edge above node n in T (or zero, if n has no parent), initialize $k = 1$, $x(n) = 0$, and $v(n) = 0$ for all $n \in \mathcal{N}$. At all times, define $z(n) = \sqrt{\ell_{T_k}(n)(b_k - x(n))}$. The k-th tree T_k has span $s_{T_k} = b_k - b_{k-1}$.

T1. [Remove edges] For each edge $(c, p) \in R_k$, and for each node $n \geq_T p$, sample $\mathbf{w}_n \sim \text{Gaussian}(0, 1)$; set $v(n) \mathrel{+}= z(n)\mathbf{w}_n$; then set $v(c) \mathrel{+}= v(n)$ and $x(n) = b_k$. Then, set $x(c) = b_k$ and remove the edge.

T2. [Add edges] For each edge $(c, p) \in R_k$, and for each node $n \geq_T p$, sample $\mathbf{w}_n \sim \text{Gaussian}(0, 1)$; set $v(n) \mathrel{+}= z(n)\mathbf{w}_n$; then set $v(c) \mathrel{-}= v(n)$ and $x(n) = b_k$. Then, set $x(c) = b_k$ and add the edge.

T3. [Iteration] If $k < K$, set $k = k + 1$ and return to **T1**. Otherwise, set $g_s = v(s)$ for $1 \leq s \leq n_S$ and finish.

The algorithm proceeds by storing and updating intermediate quantities v, z, and x indexed by nodes. The genetic value is recovered at the end of the algorithm by aggregating these intermediate values efficiently. Updating the intermediate values is also efficient because it only alters the entries that are ancestral to the added or deleted edge in the local tree while leaving all other entries constant.

To prove that the algorithm works, we first reformulate the algorithm so that we have the edge effects beforehand, making the algorithm deterministic. To this end, for each node n in the kth tree, let $\mathbf{z}_{n,k}$ have a Gaussian distribution with mean zero and variance equal to the span of the tree, $b_k - b_{k-1}$. Recall that $x(n)$ is the last position at which node n was visited, and so we can define $k(n)$ so that $b_{k(n)} = x(n)$. Now, define $Z(n) = \sqrt{\ell_{T_k}(n)}(\mathbf{z}_{n,k(n)} + \cdots + \mathbf{z}_{n,k})$. Comparing to Algorithm T, $Z(n)$ and $z(n)\mathbf{w}_n$ have the same distribution; the only difference is that we sample from the Gaussian here in several steps. Thus, where steps **T1** and **T2** say "set $v(n) \mathrel{+}= z(n)\mathbf{w}_n$", instead replace these with the equivalent instruction "set $v(n) \mathrel{+}= Z(n)$".

To show that the algorithm calculates the genetic value, we first show how to obtain the genetic value for each node based on the genome up until the current position. Then, we show that steps **T1** and **T2** in Algorithm T do not change the genetic value.

First, define the "working genetic value", which is the genetic value of node n calculated only the genome up until position b_k, as

$$g_k(n) = \sum_{h=1}^{k} \sum_{u \geq_T n} \sqrt{\ell_{T_h}(u)}\mathbf{z}_{u,h}.$$

This has the correct distribution because $\sqrt{\ell_{T_h}(u)}\mathbf{z}_{u,h}$ has a Gaussian distribution with mean zero and variance equal to $(b_h - b_{h-1})\ell_{T_h}(u)$, which is the area of the edge above u in tree h. By additivity of Gaussians, the contribution of a given edge that spans multiple trees is again Gaussian has mean zero and variance equal to its area.

Also, define

$$\hat{g}(n) = \sum_{r : r \geq_T n} (v(r) + Z(r)). \tag{A16}$$

We claim that at any point in the algorithm,

$$g_k(n) = \hat{g}(n), \qquad \text{for all } n. \tag{A17}$$

At the end of the algorithm, the value returned is $\hat{g}(s)$, because tree is empty at termination, so the right-hand side reduces to $v(s)$. $z(s)$ is zero because $x(s) = b_K$ (recall that $z(n) = \sqrt{\ell_{T_k}(n)(b_k - x(n))}$). Therefore, if equality (A17) holds throughout the algorithm, then $v(s)$ is the desired genetic value, $g_K(s)$.

We prove equation (A17) by induction. The equality trivially holds at the beginning of the algorithm, because both sides are zero. Next we prove that each of the three steps in the algorithm preserves the equality (A17).

Step T3 is the easiest to analyze. By the induction step, we assume that we began this step with $\hat{g}(n) = g_k(n)$. The current tree T and the intermediate quantities v and x do not change. Advancing the position from k to $k' = k + 1$ changes $Z(n)$ by adding $\sqrt{\ell_{T_k}(n)}\mathbf{z}_{n,k+1}$, and so changes $\hat{g}(n)$ by

$$\sum_{r : r \geq_T n} \sqrt{\ell_{T_k}(r)}\mathbf{z}_{r,k+1}.$$

Comparing to equation (A16), this is equal to $g_{k+1}(n) - g_k(n)$, and so at the end of this step, $\hat{g}(n) = g_{k'}(n)$, as desired.

This is the only step where the working genetic value changes. In the other two steps, v, z, and x change, but $\hat{g}(n)$ remains constant, thus preserving equation (A17). We prove this only for **T2**; the argument is basically the same for **T1**.

Suppose that we added edge (c, p) where c and p are child and parent nodes of the edge, respectively. Call the tree at the start of the step T, and the tree after the step T', and similarly for x', v', Z', and $\hat{g}'$. Since adding an edge only increases the set of nodes above n, the difference in $\hat{g}(n)$ caused by **T2** is

$$\begin{aligned}
&\hat{g}'(n) - \hat{g}(n) \\
&\quad = \sum_{r : n \leq_{T'} r} (v'(r) + Z'(r)) - \sum_{r : n \leq_T r} (v(r) + Z(r)) \\
&\quad = \sum_{r : n \leq_{T'} r, n \not\leq_T r} (v'(r) + Z'(r)) \\
&\quad + \sum_{r : n \leq_T r} (v'(r) + Z'(r) - v(r) - Z(r)).
\end{aligned} \tag{A18}$$

We can divide the nodes of $\mathcal{N}$ into four categories as (1) the child node c, (2) nodes below c, (3) the parent node p and nodes above p, and (4) all other nodes. Since $v(r)$ and $Z(r)$ only change for nodes that are above c in T', evidently $\hat{g}'(n) = \hat{g}(n)$ for nodes in the last category. Next, note that $v(n) + Z(n) = v'(n) + Z'(n)$ for nodes n that are above p: this is because **T2** first sets $v'(n) = v(n) + Z(n)$, then sets $x(n) = b_k$, which has the effect that $Z'(n) = 0$. Therefore, $\hat{g}'(n) = \hat{g}(n)$ for nodes $n \geq_T p$.

This leaves only nodes $n \leq_{T'} c$. The first term in equation (A18) sums over $\{r : n \leq_{T'} r, n \not\leq_T r\}$: nodes that are above n after adding the edge, but were not above n before the

addition. Hence, for nodes below c, this set is simply all nodes above p, and the first summation is

$$\sum_{r:p\leq_{T'}r} (v'(r) + Z'(r)). \tag{A19}$$

The only nodes that change x are those above p, so for nodes $r \leq_T c$, $Z'(r) = Z(r)$. Furthermore, $v'(r) = v(r)$ for nodes strictly below c. Thus, the summand of the second summation is $v'(r) + Z'(r) - v(r) - Z(r)$ is equal to 0, except for $r = c$, in which case it is $v'(c) - v(c)$. Therefore, the second summation is just $v'(c) - v(c)$. Now, $v(c)$ changes because this step, for each $r \geq_T p$, first sets $v'(r) = v(r) + Z(r)$, and then subtracts $v'(r)$ from $v(c)$. As a result, still for nodes n below c, the second summation is

$$v'(c) - v(c) = -\sum_{r:p\leq_T r} (v(r) + Z(r)). \tag{A20}$$

Putting these together, the two summations in equation (A18) cancel, so $\hat{g}'(n) = \hat{g}(n)$ for all remaining nodes.

In summary, **T1** and **T2** modify the intermediate values v, x, and Z while leaving the working genetic value constant so that **T3** correctly updates the genetic value. See **Efficient ARG-LMM simulation** for more details of fully simulating the trait based on this algorithm.

Submatrix computation

`tskit` provides multiplication by $\mathbf{B}$ (and $\mathbf{B}^{-1}$ via conjugate gradient), and for its submatrices, one can multiply an incidence matrix before and after multiplying $\mathbf{B}$ to perform matrix-vector multiplication. For instance, $\mathbf{B}_{n,o}\mathbf{v}$ can be done by:

$$\mathbf{B}_{n,o}\mathbf{v} = \mathbf{A}_n \cdot \mathbf{B} \cdot \mathbf{A}_o^T \mathbf{v}, \tag{A21}$$

where $\mathbf{A}_n$ and $\mathbf{A}_o$ are incidence matrices with each row corresponding to the non-phenotyped and observed individuals. For each row, only one column that corresponds to the index of the row in the full list of individuals are set to 1 and 0 otherwise. Multiplication by incidence matrices $\mathbf{A}_\bullet$ can be done very quickly as they are very sparse.

The conditional variances of the predicted genetic values may be useful as a measure of precision for individual BLUPs, but in large-scale applications assembling the full covariance matrix (equation 20) is infeasible. However, we can obtain unbiased estimates of the diagonal using the XDiag algorithm in Epperly *et al.* (2024), which only requires matrix-vector products of equation (20) against a fixed number of random test vectors.
